# The New Hampshire Journal of Medicine

**Published:** 1850-09

**Authors:** 


*The New Hampshire Journal of Medicine.* Edited by EDWARD H. PARKER, A. M. M. D.—This is the title of a new Medical Journal of thirty-two pages, the first number of which was issued in August. It is to be published monthly, at Concord, N. H. The subscription price one dollar per annum.

